# Unravelling the Potential of Graphene Quantum Dots in Biomedicine and Neuroscience

**DOI:** 10.3390/ijms21103712

**Published:** 2020-05-25

**Authors:** Giordano Perini, Valentina Palmieri, Gabriele Ciasca, Marco De Spirito, Massimiliano Papi

**Affiliations:** 1 Department of Neuroscience, Università Cattolica del Sacro Cuore, 00168 Roma, Italy; giordano.perini@unicatt.it (G.P.); gabriele.ciasca@unicatt.it (G.C.); marco.despirito@unicatt.it (M.D.S.); 2 Fondazione Policlinico Universitario A. Gemelli IRCSS, 00168 Roma, Italy

**Keywords:** graphene, quantum dots, blood brain barrier, theranostics

## Abstract

Quantum dots (QDs) are semiconducting nanoparticles that have been gaining ground in various applications, including the biomedical field, thanks to their unique optical properties. Recently, graphene quantum dots (GQDs) have earned attention in biomedicine and nanomedicine, thanks to their higher biocompatibility and low cytotoxicity compared to other QDs. GQDs share the optical properties of QD and have proven ability to cross the blood-brain barrier (BBB). For this reason, GQDs are now being employed to deepen our knowledge in neuroscience diagnostics and therapeutics. Their size and surface chemistry that ease the loading of chemotherapeutic drugs, makes them ideal drug delivery systems through the bloodstream, across the BBB, up to the brain. GQDs-based neuroimaging techniques and theranostic applications, such as photothermal and photodynamic therapy alone or in combination with chemotherapy, have been designed. In this review, optical properties and biocompatibility of GQDs will be described. Then, the ability of GQDs to overtake the BBB and reach the brain will be discussed. At last, applications of GQDs in bioimaging, photophysical therapies and drug delivery to the central nervous system will be considered, unraveling their potential in the neuroscientific field.

## 1. Introduction

Since their first synthesis in the early 1980s [1], Quantum Dots (QDs) have been characterized, studied and applied in various devices, such as solar cells [2] and optical devices [3].

More recently, the interest in QDs has widely spread towards different branches of biology and medicine thanks to their photophysical properties [4], which make them excellent candidates for use in bioimaging, drug delivery and, importantly, in theranostic applications such as photothermal therapy.

**Figure 1:**
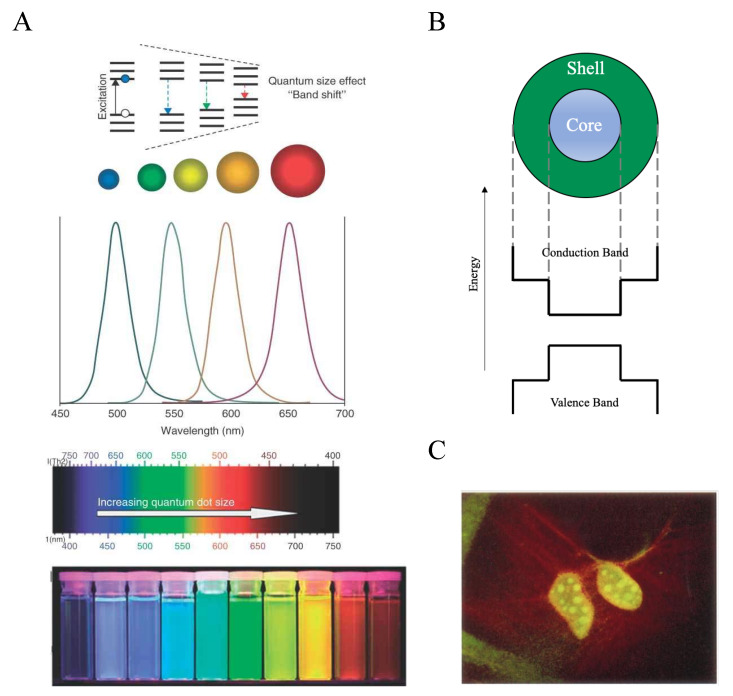
Properties of quantum dots. (**A**) Correlation between optical properties and the size of quantum dots. Reproduced with permission [5]; (**B**) Typical structure of quantum dots with a central core in which electrons occupy only discrete energy levels, and an insulator shell; (**C**) 3T3 mouse fibroblasts labeled with cadmium selenide/cadmium sulfide quantum dots (CdSe-CdS QDs) [12]. Reproduced with permission from American Association for the Advancement of Science.

Quantum dots are semiconducting nanomaterials with dimensions below 100 nm [5]. Physical and chemical properties of QDs strictly depend on their size, due to quantum confinement effect [6]. Briefly, when an electron is promoted to the conduction band from the valence band, it leaves an empty electron state, a “hole”. Electrons and holes are attracted to each other by electrostatic Coulomb force, resulting in a bound state, called excitons [7], which are neutral quasiparticles. Since QDs have dimensions comparable to the exciton diameter, they can be treated as particles-in-a-box, in which the energy can be expressed as [8]:

$$E=\frac{h^{2}\pi^{2}}{2mL^{2}}$$

where *h* is the reduced Planck constant, *m* is the mass of the particle and *L* is the length of the box. In QDs, this leads to a dependence of the bandgap energy on the size: the smaller the size, the bigger the bandgap energy (Figure 1A). This becomes evident by measuring their variations in absorption and emission as a function of the decreasing size, called “blue-shifts”, which indicate an increase in bandgap energy [5]. In QDs, as in the particle-in-a-box, electrons can occupy only certain discrete energy levels, which earned them the name of “artificial atoms” [9].

QDs have unique characteristics compared to organic fluorophores, one of which is the broader absorption spectra, enabling excitation by a wide range of wavelengths [10], and narrower emission spectra, which reduces signal overlap. Synthesis methods to produce QDs mainly involve surface passivation of an inner layer (core) by deposing a capping layer (shell), which is an inorganic semiconductor material. The core layer has a narrower bandgap than the shell. Synthesis of QDs usually occurs, in a coordinating solvent, in the presence of a core material from the 16th group (typically S or Se) and of an organometallic shell precursor (like Zn or Cd) at high temperatures (Figure 1B) [11].

QDs have also very stable light emission, which makes them less susceptible to photobleaching than other molecules. This has been outlined in many biological labeling experiments where the photostability of QDs was compared with commonly used fluorophores [10]. To demonstrate this feature, Bruchez and coworkers used, for the first time, cadmium selenide/cadmium sulfide (CdSe-CdS) QDs to label mouse fibroblasts [12]. In their experiment, biotin was covalently bound to the surface of the nanocrystals. The conjugated molecule was used to specifically mark F-actin on the cells (Figure 1C). In this way, they established a class of fluorescent probes with a long fluorescence lifetime and higher tunability. 

Following this experiment, different studies started to focus on the production of biocompatible QDs [13], on their labeling of biological samples [14] and on the use of QDs as drug delivery systems, both with or without functionalization with specific target molecules [15,16]. Bao and colleagues produced cadmium telluride (CdTe) QDs by a novel synthesis strategy. They incubated S. Cerevisiae with salts of Cd and Te, and the yeast was able to produce extracellular nanocrystals of CdTe with size-dependent emission [13]. Nanoparticles showed very high biocompatibility thanks to the natural spontaneous capping of biological macromolecules surrounding them. Zinc Oxide (ZnO) QDs surface functionalized with a biocompatible capping agent (3-aminopropyl trimethoxysilane) and were synthesized to provide potential applications in in vivo bioimaging and cancer detection [14]. ZnO is more biocompatible than Cd, since it is already present in biological systems. Furthermore, Zinc Selenide (ZnSe), ZnS, CdTe, CdS and CdSe QDs were used for different biomedical applications, such as tumor imaging, monitoring of extracellular and intracellular trafficking and nanoparticle-mediated drug delivery [15]. These nanoparticles were surface modified with biomolecules to avoid potential toxic release of shell atoms [11].

As the popularity of QDs started growing, concerns about their cytotoxicity began to rise [17,18]. The main concerns were about the inherent toxicity of the core elements of the QDs, such as cadmium and selenium, that could affect both cell cultures and live animals [18,19].

In the last years, graphene quantum dots (GQDs) have been showing great photophysical properties as well as good biocompatibility [20], having more “molecule-like” character than other QDs [21], thus spreading in life sciences applications.

Graphene is a bidimensional material, a single-atom-thick sheet of honeycomb-arranged, sp^2^-bonded carbon atoms. Graphene and graphene oxide (GO) have been employed in several biomedical applications, as antibacterial agents [22,23,24], scaffolds for bone regeneration [25,26] and as diagnostic tools [27,28]. Despite the wide range of applications of graphene and GO in the biomedical field and their biocompatibility, the use of GQDs comes with further advantages. GQDs, as QDs, have unique optical properties which strictly depend on their size, shape and surface chemistry, making them highly suitable for bioimaging compared to other organic dyes [12,21]. Furthermore, the small dimensions of these nanoparticles easily allow them to cross biological barriers and target specific anatomical regions inaccessible for graphene and GO, making them good candidates for drug delivery.

In this review, the optical properties of GQDs will be described. Then, an overview of synthesis methods will be briefly discussed. To point out their high suitability in biomedical applications, the current state of the art, including toxicity reports will be reported. We will discuss their capability of crossing the blood-brain barrier and how this capability has been used for bioimaging, phototherapy and drug delivery to the central nervous system (CNS).

## 2. Graphene Quantum Dots

**Figure 2:**
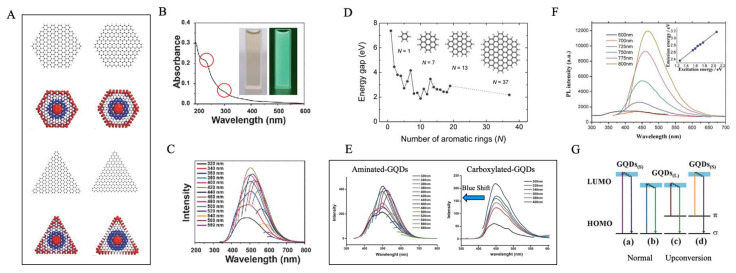
Graphene quantum dots’ optical properties. (**A**) Hexagonal and triangular-shaped graphene quantum dots (GQDs) in two distinct conformations: armchair (left) and zigzag (right), and their relative charge distribution (negative in red and positive in blue). Adapted with permission [6]; (**B**) Typical absorption peaks of GQDs at 230 and 300 nm. Adapted with permission from The Royal Society of Chemistry [31]; (**C**) Photoluminescence (PL) intensity at different excitation wavelengths. Adapted with permission from The Royal Society of Chemistry [31]; (**D**) Reduction of energy gap (red shift) as a function of sp^2^ domains. Reproduced with permission [34]; (**E**) Influence of functional groups on PL properties. Amination increases quantum yield, while carboxylation causes blue shifts due to the clusterization of sp^2^ domains. Adapted with permission [38]; (**F**) Upconversion of GQDs, showing a reduction in emitted wavelength at higher excitations [41]; (**G**) HOMO-LUMO transitions for big GQDs (b,c) and smaller GQDs (a,d). **F** and **G** were reproduced with permission from The Royal Society of Chemistry [41].

GQDs are small graphene flakes (<100 nm) in which the quantum confinement of excitons becomes dominant [21], causing a random spacing of Coulomb blockade peaks rather than a periodic distribution [6]. Interestingly, in GQDs, quantum confinement is not given only by the size, since different boundaries lead to diverse spin polarization and energy spectrum (Figure 2A). Indeed, other factors involved in quantum confinement are related to the chirality and shape of graphene edges [6,29], thus it strictly depends on GQDs’ synthesis method. 

GQDs have absorption peaks that are comparable with those of other graphene-based materials. In particular, GO shows two main peaks: one around 230 nm, and one red-shifted at 300 nm [30,31] (Figure 2B). These two distinct peaks depend on two specific electronic transitions: the first is determined by $\pi -\pi^{*}$ transitions within the aromatic rings and, in general, by the sp^2^-hybridized portions. The second, less intense, peak is due to $\pi -\pi^{*}$ transitions, and it is given by the presence of lone pairs within oxygens. As evidence, pristine graphene displays only a $\pi -\pi^{*}$ transition peak at 270 nm, and no $n-\pi^{*}$. GQDs, in turn, have a first absorption peak between 200 and 270 nm, and one at wavelengths greater than 280 nm, which occur within $\pi -\pi^{*}$ and $n-\pi^{*}$ transitions, respectively [32]. The main features which determine the absorption and photoluminescence (PL) properties of graphene-based materials involve the size of the molecule, its sp^2^-hybridized fraction, the presence of functional groups containing, in particular, oxygen or nitrogen, and the relative synthesis method [33]. As mentioned above, size plays a crucial role in the PL of GQDs. The dimension of GQDs is comparable to the exciton diameter, so they can be treated as particles-in-a-box, for which the bandgap energy is inversely proportional to the squared size. Higher bandgap energies correspond to smaller emission wavelengths [8]. Accordingly, it has been reported that the absorption peak of GQDs with lateral dimensions ranging from 1 to 4 nm is located at 270 nm, while 7–11 nm GQDs showed a maximum located at 330 nm. GQDs of 60 nm, conversely, had a peak at 280 nm [30]. This turnaround could easily be explained by taking into account the amount of sp^2^ structures within the molecule. As discussed by Eda and colleagues, in carbon materials containing a mixture of sp^2^ and sp^3^ bonding, the opto-electronic properties are determined by the π states of the sp^2^ sites, which are embedded in the consistently higher bandgap of σ and $\sigma^{*}$ orbitals [34]. Therefore, recombination of electron-hole pairs in sp^2^ clusters results in PL (Figure 2C). In order to tune PL emission, the amount and nature of sp^2^ sites can be controlled, since bandgap depends not only on shape and size, but also on the fraction of sp^2^ domains (Figure 2D). Eda measured the energy gap of carbon materials as a function of the number of sp^2^ aromatic rings. A single aromatic ring (benzene) showed a bandgap of 5.4 eV. With the increase of the number of aromatic rings, a progressive reduction in energy gap occurred. GO with 20 aromatic rings had a bandgap of 2 eV, while 100 aromatics, which had a size of around 3 nm, showed an energy gap of 0.5 eV. The higher the number of aromatic rings, the less the energy gap, and the higher the wavelength of emission [35].

Carbon materials with disordered sp^2^ clusters act as amorphous semiconductors, in which the density of states for aromatic chains falls in localized states inside the bandgap, reducing the energy gap between valence and conduction band [36]. The number of functional groups containing nitrogen contributes as well in GQDs optical properties: their lone pairs increase the second absorbance peak related to $n-\pi^{*}$ transitions [34]. Wang and colleagues [37] synthesized nitrogen-doped carbon dots with a quantum yield of 23% and an absorption spectrum peaked at 280 nm instead of the previously reported (230 nm) [33]. The increase in oxygen contributed to the formation of smaller sp^2^ regions, which were responsible for the blue shifts accordingly to the bandgap related to the reduction of aromatic rings [38] (Figure 2E). Therefore, functionalization of GQDs’ surface plays a key role in their suitability for biological applications, in particular for bioimaging and drug delivery. Other bioimaging agents, such as organic compounds or even QDs, lack tunable surface specific functionalization when compared to GQDs, since chemistry of carbon is the most studied and characterized, allowing a more refined surface engineering. Lastly, the synthesis method can affect optical properties as well. Hydrothermal methods of synthesis can decrease the average number of oxygens, increasing the number of aromatic rings, thus reducing the bandgap energy [30]. 

Many studies reported upconversion properties of GQDs [21,39,40]. Photon upconversion occurs when emitted photons have higher energy (shorter wavelength) than incident photons (Figure 2F). This phenomenon in quantum dots has been explained as an anti-stokes transition in which π orbital electrons, after being excited from the highest occupied molecular orbital (HOMO) to the lowest unoccupied molecular orbital (LUMO), go back to a σ orbital (with lower energy); therefore the resulting emitted photon has higher energy than the incident one [40,41] (Figure 2G). While the LUMO-HOMO transition appears to be a reasonable explanation for the upconversion, some studies suggest that other principles might be inherent in GQDs’ behavior. Gan and coworkers [42] and Wen and colleagues [43], raised objections on this optical property of quantum dots. They stated that shorter emission wavelengths could be due to second order diffraction through the monochromators [43].

## 3. Synthesis of GQDs

**Scheme 1:**
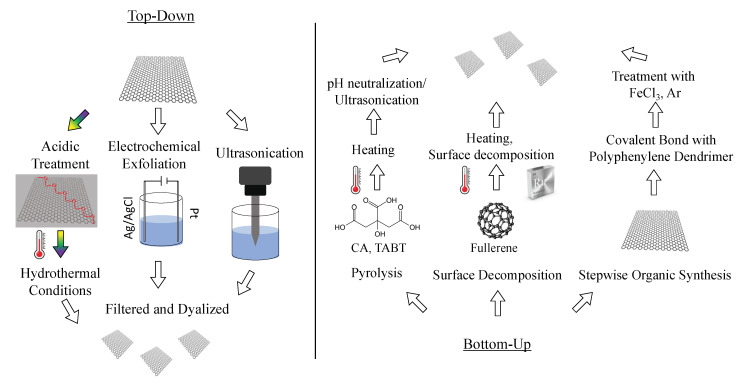
Representation of the main strategies for the synthesis of GQDs.

The main strategies of GQDs’ synthesis are reported in Scheme 1.

Synthesis methods of GQDs can be classified in top-down and bottom-up [44,45] approaches. Top-down strategies have the advantage to use cheap, easily available and bulk graphene-based carbonaceous precursors such as GO, carbon fibers, carbon black and graphite. In this approach, materials are cut into small-sized GQDs through chemical, electrochemical or physical methods [46]. In top-down methods, graphene-based materials are exfoliated and decomposed in multiple steps by concentrated acids, strong oxidizing agents and high temperatures [21].

In the hydrothermal method, GO sheets are furtherly oxidized under acidic treatment (HNO_3_), to create a line of rupture along the C–C bonds, due to the formation of epoxy groups [47]. In hydrothermal conditions (200 °C, 10 h) and alkaline medium (pH = 8), the epoxy groups are stabilized in carbonyl groups, which make the so-formed GQDs soluble in water. This procedure has subsequently been implemented by thermally reducing GO in graphene sheets (600 °C, 2 h) and by improving alkaline conditions (pH = 12), obtaining green luminescent GQDs [46]. Hydrothermally synthesized QDs can have different PL properties according to the acidic conditions used for synthesis [48]. By passivating GQDs with polyethylene glycol (PEG), a highly luminescent and up-converted dispersion of GQDs can be obtained [48]. Zhu reported a different thermally-mediated synthesis of GQDs, called solvothermal, in which dimethylformamide (DMF) was used as a solvent and reducing agent [49]. This step was followed by a chromatographic separation based on the degrees of oxidation, obtaining different PL GQDs [21].

The acidic exfoliation is one of the simplest ways to produce GQDs. In this procedure, GO is treated with strong acids, resulting in negatively charged (hydrophilic) and highly defective GQDs. One of the sources used to produce GQDs is coal [50]. Ye et al. synthesized coal starting from GO in a solution of H_2_SO_4_ and HNO_3_. GO was then sonicated for 2 h, then stirred and heated at 100 °C for 24 h. The pH was set to 7 by adding NaOH, and the solution was furtherly dialyzed to obtain a monodisperse solution of GQDs. Other sources for acidic exfoliation are carbon fibers [51] and carbon black [52], with the same chemical treatments.

The acidic exfoliation has been implemented with microwave irradiation, which provides uniform heating for the reaction medium [53]. The initial acidic exfoliation was performed in a mixture of GO, H_2_SO_4_, and HNO_3_. The mixture was then heated for 3 h, filtered and dialyzed. In this way, green and blue luminescent GQDs were obtained. Interestingly, Tang et al. used the microwave irradiation method with glucose, obtaining GQDs emitting in the deep ultraviolet (303 nm) [54].

Another top-down strategy is electrochemical oxidation. In this method, a cyclic voltammogram was applied on graphene in phosphate buffer. Oxygen plasma was used as a working electrode, while the counter electrode consisted in platinum wire. The suspension was filtered with a microporous membrane and furtherly dialyzed, obtaining green luminescent GQDs [55].

Zhou and coworkers synthesized GQDs with up-converted and down-converted emission which appeared to be independent of the excitation wavelength [40]. In this top-down procedure, GO was ultrasonicated for 12 h, filtered and dialyzed.

Bottom-up techniques provide excellent control of the properties of the final product. In these strategies, chemical reactions, like stepwise organic synthesis, are exploited to convert small organic molecules (such as citric acid, fullerene and polycyclic aromatic hydrocarbons) to high-quality GQDs. However, the main disadvantage of bottom-up techniques lies in the complexity of the procedures of synthesis [46].

The most used bottom-up method is pyrolysis. Dong prepared GQDs, using as a starting material, citric acid (CA) [56]. In this work, CA was heated to 200 °C, after 5 min CA was liquated, and after 30 min, the color of the solution changed to orange, suggesting the formation of GQDs. The suspension was then dissolved, drop by drop, in 100 mL NaOH until it reached neutral pH. GQDs obtained with this method were passivated due to the incomplete carbonization of CA, and showed a blue PL. Li and coworkers used, as a precursor for the synthesis of nitrogen-doped quantum dots, 1,3,5-tri-amino-2,4,6-trinitrobenzene (TABT) heated in a furnace at 750 °C in nitrogen atmosphere [57]. Then, the sample was cooled down until it returned to room temperature. The so-formed N-GQDs were sonicated for 2 h and stirred with H_2_SO_4_ and HNO_3_ for 24 h at 100 °C. The pH was then tuned to 8 with Na_2_CO_3_ and dialyzed. Blue PL N-GQDs sheets of 15 nm in size were imaged with atomic force microscopy (AFM), showing a height mostly distributed in the range of 0.5–2.0 nm (single and double layers).

GQDs were also synthesized by using fullerene (C_60_) as starting material [58]. In that procedure, surface-catalyzed decomposition of fullerene on reactive transition metal (ruthenium) was used, by reaching a temperature of 1200 K. To avoid the formation of carbon aggregates, low coverage of C_60_ was exploited, so that the interparticle distance was increased. In this way, GQDs with mainly triangular shape and almost no surface defects were obtained. Another group synthesized highly soluble GQDs containing 168 conjugated carbon atoms, the largest soluble GQDs reported, via stepwise organic synthesis [59]. They developed a strategy to reduce the tendency of forming insoluble aggregates by large graphene nanostructures, which consisted of covalently attaching multiple 1,3,5-trialkyl-substituted phenyl moieties to the edges of graphene.

## 4. Biocompatibility and Cytotoxicity of Quantum Dots (QDs) and Graphene Quantum Dots (GQDs)

Though research on GQDs is at a relatively early stage, their high biocompatibility and low cytotoxicity have already been demonstrated in vitro and in vivo, enhancing the interest in these nanomaterials compared to other QDs. In Table 1, we summarized studies on GQDs and QDs discussed in this review.

Concerning QDs, data on toxicity are controversial. In vitro QDs’ short-term exposition experiments were mainly carried out on cancer cell lines such as HeLa [68], or human breast cancer MDA-MB-231 [69] cell lines, which are known to be less sensitive to heavy metals. On these cells, QDs did not show significant toxicity. Conversely, Derfus and coworkers [19] used rat hepatocytes to demonstrate a high toxicity due to cadmium ions release, proportional to the degree of oxidation of the QDs [70]. In the same year, Shiohara and colleagues confirmed toxicity on Vero cells, human hepatocytes and, interestingly, on HeLa cells [17]. In vivo toxicity studies were conducted on mice with repeated intraperitoneal injection with mercaptopropionic acid-conjugated CdSe-CdS QDs at different doses every 3 days, for 15 days [60]. QDs were detected in the liver, spleen, lung and kidneys, but not in the brain and heart. A dose-dependent distribution was reported with oxidative stress measured in spleen and liver at high doses of QDs, and in lung and plasma 7 days after injection. In this work, the level of pro-inflammatory cytokines was also evaluated, showing an increase in the spleen and liver, and in the plasma samples at day 10, 13 and 15.

Another group reported accumulation of water-soluble cadmium containing QDs in mice in the liver, kidney, spleen and lung, carrying out an 80-days experiment. At 15 days post-injection, uptake in the liver decreased, while uptake in kidney greatly increased [71]. Afterward, uptake in liver or kidney was stable during this time. It was also pointed out that kidney uptake could be related to the size of QDs: when the hydrodynamic diameter was increased from 2.9 to 4.5 nm, kidney uptake decreased as well, indicating that QDs with smaller sizes are more easily absorbed by the kidney. In this in vivo toxicity study, mice survived for 80 days after intravenous injection, without evident toxic effects, differently from mice in Haque and coworkers’ study [60].

GQDs’ toxicity studies reported promising results. In a recent work, cell viability of the total population of mononuclear blood cells was investigated after incubation with GQDs, and cytotoxicity was not reported below 500 µg/mL [61]. Tomić and coworkers studied the suppression of the inflammatory response of T cells through the induction of autophagy on dendritic cells by GQDs [72]. At concentrations higher than 300 µg/mL, cell viability started decreasing, reaching approximately 50% at 500 µg/mL. Accordingly, another work has shown the effects of C. elegans nematode chronic exposure to three graphene-based materials (graphene, GO and GQDs) [62]. GQDs showed lower lethality compared to GO, while in all cases, similar neurotoxicity was pointed out. Toxicity was related to physiological parameters such as body bending and head thrashing. After exposure to graphene-based materials, they also found significant decrease in mean speed, frequency of bending and wavelength of the crawling movement, suggesting that high concentrations of these nanomaterials could damage dopaminergic and glutamatergic neurons.

**Figure 3:**
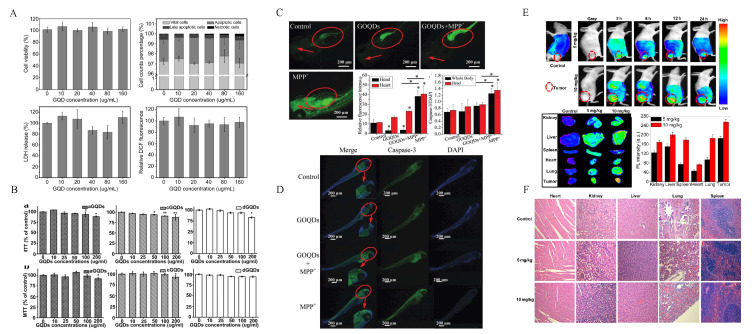
Toxicity of GQDs. (**A**) Cell viability tests and reactive oxygen species (ROS) production on HeLa cells. Reprinted from [64]. Copyright 2014, permission from Elsevier; (**B**) Cell viability on A549 (top) and C6 (bottom) after the treatment with aminated GQDs (aGQDs), carboxylated GQDs (cGQDs) and DMF GQDs (dGQDs). Reproduced with permission [38]; (**C**) Oxidative stress on zebrafish after the treatment with MMP and GQDs with relative fluorescence intensity on head and heart. Reprinted from [65]; (**D**) Fluorescence intensity of caspase-3 after the treatment with GQDs and MMP [65]; (**E**) In vivo and ex vivo imaging of mouse after injection with carboxylated GQDs [67]; (**F**) Accumulation of different concentrations of carboxylated GQDs in mouse organs, 160× magnification. **E** and **F** were reprinted with permission [67]. Copyright 2013, American Chemical Society.

Furthermore, while these findings demonstrated a correlation with concentration and exposure time, other works highlighted a dependence of cellular viability on the presence of specific functional groups on GQDs. Xu and colleagues have demonstrated aminated GQDs’ (NH_2_-GQDs) potential role as nuclear markers in rat alveolar macrophages [63]. NH_2_-GQDs’ cytotoxicity at relatively low concentrations (<200 µg/mL) was also studied. Chong and coworkers investigated the toxicity of GQDs in vitro, on HeLa cells (Figure 3A), and in vivo, by measuring PL intensity and weight indexes of mice’s organs at different timepoints after the injection of GQDs or GO, showing fast clearance and no toxicity [64]. Yuan and coworkers focused on the synthesis of three different types of functionalized GQDs with -NH_2_, -COOH and -DMF groups [38]. After the synthesis, they compared the relative cytotoxicity and observed their cellular distribution in human A549 lung carcinoma cells and human neural glioma C6 cells. A significant proliferation decrease was induced by aminated and DMF GQDs when the concentration reached 200 μg/mL (Figure 3B). When the concentration of carboxylated GQDs reached 50 μg/mL, cell viability was statistically different from the control. Similar results were obtained on C6 cells. However, even at the highest concentrations, more than 80% of the cells remained vital, demonstrating how GQDs with different functional groups possess low cytotoxicity, in contrast with Xu results.

Ren and colleagues demonstrated a reduction in apoptosis and oxidative stress mediated by 1-methyl-4-phenyl-pyridinium ion (MPP+) when PC12 cells were treated GQDs, thus showing neuroprotective effects of these nanomaterials [65]. Neuroprotection was furtherly confirmed in the same work in vivo on zebrafish (Figure 3C,D). The treatment with GQDs and MMP decreased both caspase-3 expression (apoptosis), as well as the oxidative stress, when compared to control and zebrafish treated with MMP only. Another group focused on the effects of functionalization of GQDs with carboxyl residues both in vitro and in vivo [66]. In vitro studies were carried out on different types of cells: human breast adenocarcinoma MDA-MB-231, KERATIN-forming tumor KB cells, A549 and Madin-Darby Canine Kidney MDCK cells after treatment with carboxylated GQD at 50, 100, 250 and 500 μg/mL concentration. Results were proportional to the concentration of GQDs but no acute toxicity or morphological changes were noted. In vivo experiments were conducted to investigate GQDs’ biodistribution and imaging in mice. Nanoparticles were administered to tumor-bearing Balb/c nude mice intravenously at 5 or 10 mg/kg. After 12 h, fluorescence signals were observed in vivo at the tumor site (Figure 3E). After 24 h, no signal was measured from the tumor or other regions of the body. Dissected organs and ex vivo images showed an increase after 2 h of GQDs’ concentration in the liver and heart of mice and a rapid decrease over time. After 12 h, the concentration of particles in kidneys started to slightly increase, suggesting a mechanism of rapid clearance of GQDs. Ex vivo PL intensity at 24 h post-injection was lower than that at 12 h, which is likely due to the rapid excretion of GQDs. Nurunnabi and coworkers investigated changes in hematological factors after GQDs’ administration, hypothesizing that their size comparable to viruses and small proteins could cause inflammatory responses [67]. The values of parameters such as hemoglobin concentration, white blood cells, hematocrit, and platelet count, were measured and found to be within normal ranges and were similar with control.

Taken together, these findings strongly suggest that GQDs possess very high biocompatibility and low cytotoxicity in vivo compared to QDs. 

## 5. Overtaking the Blood-Brain Barrier (BBB) with GQDs

**Figure 4:**
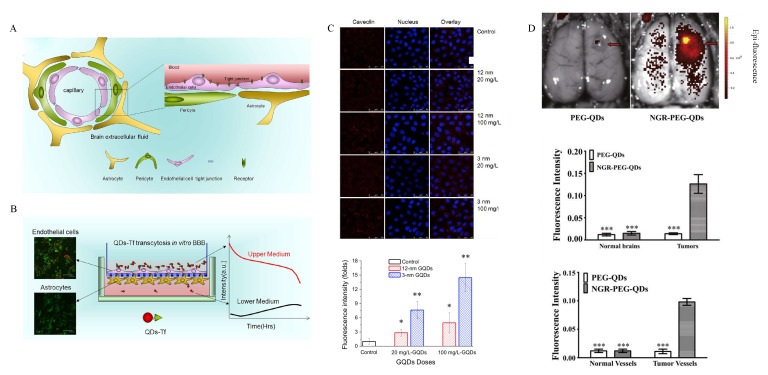
GQDs across the blood-brain barrier (BBB). (**A**) Structure of the BBB [75]; (**B**) In vitro permeability via transwell in a co-culture system. **A** and **B** were reprinted with permission [75]. Copyright 2008, American Chemical Society; (**C**) Transcellular transport mechanism across a biological barrier made of canine kidney MDCK cells with different sizes of GQDs. Scalebar 50 µm. * was for P < 0.05, ** was for P < 0.01, and *** was for *p* < 0.001 over control. Reproduced with permission from The Royal Society of Chemistry [73]; (**D**) Uptake of PEG-GQDs functionalized with a ligand of CD13, capable of recognizing glioma and tumor vessels. *** was for *p* < 0.001 over control. Adapted from [84]. Copyright 2017, permission from Elsevier.

The key element that makes the use of GQDs in the biomedical and neuroscientific fields crucial, is the increasing amount of evidence indicating their capability of crossing biological barriers [73]. In particular, the greatest challenge to date for overtaking biological barriers is represented by the blood-brain barrier (BBB) (Figure 4A). From an anatomical point of view, the BBB is constituted by an inner layer of endothelial cells with tight junctional complexes, pericytes, astrocytes and neurons. The interaction of these cellular populations is called neurovascular unit [74]. The BBB works as a critical checkpoint between the CNS and the bloodstream. On one hand, BBB prevents harmful substances from entering the brain and, on the other hand, provides the essential nutrients for neurons. The restrictive nature of the BBB represents an obstacle for drug delivery to CNS [75]. Indeed, the BBB can be considered as a dynamic interface that controls the influx and efflux of a large variety of substances, including potentially useful drugs for a wide range of brain diseases, thus making most of the current therapies ineffective [76,77]. Evidence has demonstrated that less than 2% of small-molecule drugs can cross the BBB, though these drugs possess a characteristic molecular weight below 400 Da [75] and are specifically surface-modified for the uptake [78]. Due to the inability to adequately deliver therapeutic agents across the BBB, current treatments for CNS diseases remain limited.

Several QDs have been designed to modulate or bypass the BBB for the delivery of therapeutics (Table 2). Xu and coworkers used transferrin-bioconjugated quantum rods to pass through an in vitro model of the BBB with endothelial cells and pericytes [79] (Figure 4B). These two cellular populations were cultured on a semiporous membrane, and by laser scanning confocal microscopy where the authors measured an increase in the fluorescence intensity due to QDs in the lower medium, corresponding to a decrease in the fluorescence of the upper layer. In neuroinflammatory conditions, the disruption of the integrity of the BBB is mediated by metalloproteinases. In particular, the MMP-9 causes the proteolytic degradation of the extracellular matrix, together with the activation of inflammatory leukocytes and the apoptosis of neurons. Conjugation of a short interfering RNA (siRNA) with QDs was used to reduce the expression of MMP-9 [80]. By this regulation, an increase in the synthesis of matrix proteins like collagen was observed. To measure the structural integrity of the BBB, the trans-endothelial electrical resistance (TEER) in endothelial cells treated with siRNA and with the nanoplex of QDs and siRNA was evaluated. An increase in the electrical resistance was measured for cells treated with siRNA-QDs. Wang and colleagues prepared GQDs of two different sizes (3 and 12 nm) by sonicating carbon fibers in H_2_SO_4_ and HNO_3_ [73]. Their study aimed to measure the permeability of GQDs on MDCK cells and their transcellular transport mechanism (Figure 4C). These particles were administered at different increasing concentrations up to 400 mg/L. Cell viability was kept under control for the duration of the experiment, and only a mild reduction was observed. The apparent permeability (P_app_) was higher for smaller QDs. By inhibiting the formation of lipid raft via methyl-β-cyclodextrin, Wang found a reduction in GQDs’ permeability for both sizes of GQDs, thus pointing out the relevance of this transport pathway. In 2014, Hanada and colleagues measured in vitro BBB permeability to nanoparticles having different sizes and surface chemical composition [81]. They evaluated P_app_ of silica nanoparticles with different sizes, ranging from 30 up to 400 nm, on a blank membrane (i.e., without cells) and on a commercially available BBB model. A significant higher permeability was measured for 30 nm silica particles on the BBB model, while the blank membrane did not show permeability differences with respect to the size. To evaluate the dependence of membrane permeability to the surface chemical composition of the nanoparticles, Hanada and coworkers used QDs modified with anionic (-COOH), cationic (-NH_2_) and neutral (-PEG) functional groups, having a size similar to silica particles (30 nm). They demonstrated that cationic QDs had a higher permeability capacity. NH_2_-QDs were transported onto the basolateral side through the BBB model with higher permeability than anionic or neutral QDs. This result was probably caused by surface charge; indeed as aminated particles interact with the cell surface, which is composed of anionic phospholipids, they could be transported through the BBB via the paracellular pathway, while the carboxyl particles are known to be mainly incorporated into cells by endocytosis. The evidence of Hanada were in accordance with the study of Zhang and Monteiro-Riviere [82], where cellular uptake of human keratinocytes for three different QDs with diverse surface chemistries (-NH_2_, -PEG and -COOH) was investigated. Uptake was measured only for COOH QDs indicating possible interactions between anionic QDs and cell membranes. By studying intracellular translocation pathways, authors discovered that QDs were internalized by endosomes in a microtubule-depending manner. In their proposed mechanism, the uptake of COOH QDs was mediated by both lipid rafts/caveolae and G-protein coupled receptors/low-density lipoprotein receptors, thus explaining the lower diffusion of COOH QDs observed by Hanada and colleagues in their BBB model. Lu and coworkers used aminated carbon dots synthesized by hydrothermal method in the presence of polyethylenimine [83]. They tested their dots on an in vitro model of BBB made of primary rat microvascular endothelial cells and astrocytes in a transwell. They measured the fluorescence intensity in the lower chamber at different times, pointing out an increased translocation of aminated carbon dots which was concentration- and time-dependent. An in vivo study of the BBB, conducted by Li and colleagues, reported no capability of biocompatible carbon dots to cross the barrier of a zebrafish [78]. However, by conjugation with transferrin, fluorescence intensity of the dots was measured inside CNS. In a recent work, Huang and colleagues performed in vitro and in vivo studies of uptake of PEGylated QDs functionalized with asparagine-glycine-arginine peptide, capable of recognizing CD13, an overexpressed receptor on the surface of glioma cells [84]. They tested this QD on two different in vitro models: a transwell-BBB model made of brain capillary endothelial cells (BCECs) and astrocytes, and a BTB (blood-tumor barrier) with BCECs and C6 glioma cells. They found a high increase in the fluorescence intensity in the BTB model treated with functionalized QD, while BCECs and astrocytes of their BBB did not show significant uptake of the nanoparticles. They also demonstrated that tumor-associated microvessels were labeled with the QDs, as well in the BTB. In vivo tests focused on the uptake and labeling capability of QDs across the BBB inside the induced tumor in mice (Figure 4D). 8 h after the injection, they measured a specific increase in the fluorescence intensity in the tumor region for the functionalized QDs, thus indicating their crossing and labeling ability. Thanks to their capability of crossing biological barriers, these QD-related nanotechnologies could lead to a novel prospect for the bioimaging-based molecular diagnosis and neurosurgery, drug delivery and photothermal therapy applications on brain cancers and diseases in neuroscience.

## 6. Bioimaging and Neuroimaging with GQDs

Optical properties are one of the key features of GQDs [30]. To date, most of the fluorophores used for bioimaging are organic dyes. However, they suffer from photobleaching, not allowing long- term exposure to excitation sources. Therefore, GQDs can be considered as a new class of fluorophores [16]. A wide range of studies reported labeling capabilities of GQDs in vitro and in vivo. Kumar and colleagues synthesized highly biocompatible green functionalized GQDs with a size distribution between 3 and 14 nm obtained through acidic treatment of graphite powder [85]. Ma and coworkers developed a method for the synthesis of doped and undoped GQDs, based on direct carbonization of organic precursors at solid-state [86]. In that study, they employed different precursors such as ethylene diamine tetraacetic acid (EDTA), glucose, sucrose, tartaric acid, arginine, lysine and adenosine 5′-triphosphate disodium salt (ATP), to obtain N-, S-, P- and O-doped GQDs. To show the effect of chemical doping, plain undoped GQDs synthesized from citric acid were used as a reference. The uptake of GQDs was observed from the strong fluorescence signal in cells in RAW 264.7 (mouse leukemic monocyte macrophage cell line) cells. Most of the fluorescent signal was localized in the cytoplasm of the cells. Interestingly, most of the intracellular GQDs were not in the lysosome, indicating that the uptake was not related to the endocytic processes, but more likely to a breakage of cell membrane at regions of endocytosis [82,87]. Chemical-doped GQDs with S and N were synthesized by Qu and colleagues [88,89]. Using water as a solvent for the synthesis of GQDs, they created nanoparticles with a blue emission, while the polar aprotic solvent DMF allowed an increase of nitrogen units in the GQD framework and caused a red shift of the emission peak. Another strategy in this study was based on doping GQDs with sulfur and nitrogen, creating GQDs with three different absorption and emission peaks: the blue emission associated to a chromophore containing a C=O group, and red or green emission related to chromophores containing C=N or C=S groups, respectively. In both cases, A549 cells were successfully labeled with GQDs at a concentration of 100 g/mL. Sulfur-doped GQDs with a broad absorption and great emission properties in HepG2 cells were synthesized by Sangam and colleagues [90]. HeLa cells were stained with small-sized red GQDs, containing a large number of sp^2^ regions, associated to high emission wavelengths [34,91]. N-GQDs synthesized using GO and DMF as starting materials were used to label a turbid intralipid tissue phantom with two-photon fluorescence as a potential alternative probe for noninvasive detecting imaging system in living biosystems [92].

Compared to other dyes, even with near-infrared femtosecond laser excitation (NIR), GQDs showed very little photobleaching.

From the above studies, it is evident that GQDs are excellent candidates for bioimaging applications, especially for CNS, given their BBB crossing ability (see the previous paragraph).

**Figure 5:**
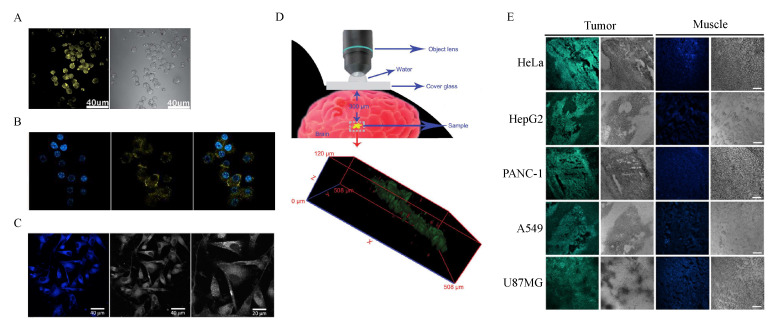
Neuroimaging with GQDs. (**A**) Confocal imaging of neurospheres with GQDs. Adapted with permission from The Royal Society of Chemistry [93]; (**B**) Confocal imaging of human neural stem cells with GQDs (green) and nuclear staining (blue). Adapted with permission from The Royal Society of Chemistry [94]; (**C**) Confocal imaging of U87 cells excited at 407 nm (blue) and 488 nm with N-doped carbon dots. Reprinted with permission [37]. Copyright 2014, American Chemical Society; (**D**) Deep tissue confocal imaging of GO nanoparticles inside mouse brain. Reproduced with permission [95]; (**E**) PH-responsive GQDs injected in mice bearing different tumors and adjacent muscles. Scale bar 40 μm. Adapted with permission from The Royal Society of Chemistry [39].

Zhang [93] and Shang [94] labeled neural stem cells with GQDs with yellow emission, due to the presence of hydrazine groups on their surface. In this study, the uptake and biocompatibility of GQDs on three different kinds of stem cells, neurosphere cells, pancreas progenitor cells and cardiac progenitor cells was studied. The uptake on neurosphere stem cells was measured by confocal microscopy, showing a high fluorescence intensity on cell membranes and within the cytoplasm, but not in the nucleus (Figure 5A). Subsequently, Shang and coworkers measured the uptake of GQDs in human neural stem cells. By measuring the fluorescence intensity with increasing concentrations of GQDs, they found the saturation point for the uptake of GQDs at 200 µg/mL. Moreover, after lysing neural stem cells, they pointed out the time dependence of the uptake. They also measured the fluorescence intensity by confocal microscopy after 24 h of incubation in three different conditions: by depletion of ATP, by incubation at 37 °C or at 4 °C (Figure 5B). The incubation at 37 °C showed the highest fluorescence intensity, indicating that GQDs can label cells in physiological conditions. By confocal microscopy, they showed how long-time exposure to GQDs caused a decrease in the relative fluorescence signal, indicating that retention of GQDs decreased after 24 h of treatment. Lastly, they demonstrated the excellent biocompatibility of GQDs on neural stem cells by showing that they did not affect the expression of stem self-renewal ability and of the cell type-specific marker (nestin). During the differentiation process, cells differentiated into neurons and glia, which supported the activity of neurons. After two weeks of differentiation, no differences were visible in the growth between the control group and the GQDs treatment group (25 µg/mL). Wang and colleagues synthesized nitrogen-doped carbon dots to label U87 human glioblastoma cells [37]. Cells were incubated with carbon dots at 20 µg/mL for 2 h, and the fluorescence imaging performance was examined by confocal fluorescence microscopy (Figure 5C). Both cell membrane and cytoplasm were labeled without invading the nucleus, and the U87 cells became brightly illuminated. Importantly, Yuan, Liu and colleagues synthesized three GQDs with diverse surface chemistries: aminated, carboxylated and functionalized with DMF [38]. They tested both labeling ability and cytotoxicity of these GQDs on C6 glioma cells. In comparison with the control cells, the fluorescence of the cells incubated with 50 μg/mL of GQDs for 12 h was brighter, which indicated the cell uptake of GQDs with different chemical groups. Most of the fluorescence intensity was raised from the cytoplasm, demonstrating that the three GQDs were not located in the nucleus. In the last years, the use of GQDs has moved to in vivo brain labeling. Qian and colleagues used GO nanoparticles of 40 nm, compatible to the size of GQDs [95]. In their study, they intravenously injected GO-PEG nanoparticles into mice, and observed their flow, distribution and clearance in the blood vessels, by utilizing two-photon imaging. They also microinjected the nanoparticles into the brain of mice expressing fluorescent oligodendrocytes, and the in vivo two-photon and three-photon luminescence imaging results showed that GO nanoparticles located at 300 mm depth could be distinguished (Figure 5D). Zheng and coworkers synthesized carbon dots from L-aspartic acid (Asp-CDs) to target CNS tumors in vivo [96]. In particular, tumor-bearing mice with C6 glioma were used. At first, they evaluated in vitro cytotoxicity and labeling ability of Asp-CDs. To verify the selectivity of carbon dots on glioma cells, C6 and mice fibroblasts were cultured and treated with the nanoparticles. Glioma cells demonstrated a higher fluorescence intensity compared to fibroblasts. To measure the ability of crossing the BBB and effectively reaching the orthotopic glioma, researchers compared Asp-CDs to CDs synthesized from l-glucose. Both nanoparticles effectively reached the mouse brain after intravenous injection. However, only Asp-CDs could specifically locate in the tumor region when compared to the other CDs, which distributed equally between brain and glioma. Ai, Ji and coworkers used carbon dots doped with manganese to perform both in vivo magnetic resonance and ex vivo optical imaging on mouse brain with tiny glioma [97]. As a result, they enhanced the contrast effect in magnetic resonance, showing their potential along with optical imaging for the detection and intra-operative location of tiny brain gliomas. Fan and colleagues [39] exploited the lower pH of solid tumors (6.4–6.8) with respect to normal tissues (7.0–7.4) [98,99]. In their work, they prepared pH-responsive GQDs capable of switching their emission from green (pH < 6.8) to blue (pH > 6.8). They used their GQDs to test whether they could distinguish the acidic environment of tumors in normal tissues. GQDs were subcutaneously injected into tumors and adjacent muscles in mice bearing different tumors, including glioblastoma multiforme. After 24 h, fluorescence microscopy images showed that tumors emitted green light, while muscles emitted blue light (Figure 5E). By intravenous injection, they tested the ability of GQDs to reach tumor regions and measured the fluorescence at different times. GQDs successfully crossed the BBB and reached the glioblastoma region. They demonstrated how GQDs have great potential to be used as a universal probe in clinic for fluorescence-guided cancer surgery and cancer diagnosis. The wavelengths reported in this review for bioimaging, phototherapy and drug delivery of neural cell lineages are summarized in Table 3 in the following paragraph.

## 7. Brain Tumor Phototherapy with GQDs

**Figure 6:**
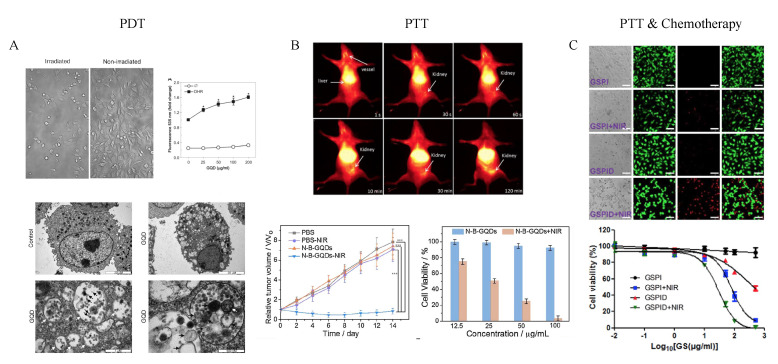
Phototherapy with GQDs. (**A**) Photodynamic therapy by irradiating U251 cells with blue light (470 nm), with relative ROS production (top) and autophagic vacuole formation (bottom, TEM). Adapted from [102]. Copyright 2012, permission from Elsevier; (**B**) NIR-II imaging (top) on xenograft C6 glioma mouse model and photothermal therapy (bottom), showing tumor growth reduction (in vivo) and loss in cell viability (in vitro). Adapted from [100]. Copyright 2019, permission from Elsevier; (**C**) Combination of PTT and chemotherapy with doxorubicin. GSPI = functionalized graphene sheets; GSPID = graphene sheets loaded with doxorubicin. Reprinted with permission [109]. Copyright 2013, American Chemical Society. Scale bar 75 μm.

Another advantage of photophysical properties of GQDs and, in particular, of their photostability, is that they can be used for cancer phototherapy. Therefore, they can be exploited for multiple applications in the theranostic field, allowing imaging and phototherapy at the same time, making them more suitable with respect to other common organic dyes for bioimaging applications [103,104]. This technique is based on two features of materials that, when exposed to light at a specific wavelength (photosensitizers), generate cytotoxic reactive oxygen species (ROS, photodynamic therapy, PDT) [105] or heat (photothermal therapy, PTT) [106], capable of killing cells by photoablation. Since photosensitizers are generally harmless without light, tumor treatment can be specifically targeted by selective illumination, limiting damage to surrounding healthy tissues. Furthermore, this therapy can be used to damage endothelial cells of tumor blood vessels. However, this emerging field lacks clinical applications, mainly due to the lack of specific photosensitizers capable of specifically targeting tumor regions [66,107]. GQDs have been used as PDT agents for the treatment of human glioma by Markovic and colleagues [102]. They tested in U251 human glioma cells, electrochemically derived GQDs capable of producing reactive oxygen species when excited with blue light. By excitation with blue light, GQDs caused both oxidative stress (Figure 6A), and an increase in early and late apoptotic cell populations, depending on the concentration and irradiation exposure time. The induction of apoptosis and autophagy in photosensitized cells is a common outcome of photodynamic therapy, to increase the clearance of oxidatively damaged organelles [108]. Markovic and coworkers found an increase in the expression of specific autophagy markers after continuous irradiation: GQDs induced oxidative stress after irradiation, and activated type I (apoptosis) and type II (autophagy) programmed cell death (Figure 6A). This feature could furtherly be exploited in PDT. Wang and colleagues produced boron and nitrogen-doped GQDs via one-pot process [100]. They used GQDs as second near-infrared window (NIR-II, with emission between 100 and 1700 nm) and analyzed in vivo and ex vivo mice bearing C6 glioma (Figure 6B). GQDs were administered intravenously, and after 24 h, fluorescence images were acquired using an excitation wavelength of 808 nm, showing that the nanoparticles could label the tumor. They then assessed the PTT behavior of GQDs, by irradiating for 5 min a solution of water containing GQDs. The temperature of pure water increased by 3 °C, whereas the temperature of water containing GQDs at concentrations of 50, 100, and 200 µg/mL increased by 11.1, 19.2, and 26.6 °C, respectively. In vitro tests showed the capability of irradiated GQDs to kill tumor cells (Figure 6B). In vivo tumor growth was stopped completely in mice with GQDs irradiated by 808 nm excitation wavelength (Figure 6B). The photothermal property of these GQDs was due to the presence of nitrogen and boron which could induce significant local distortion of electron energy and create additional energy gaps, and to the presence of defects that could significantly red-shift their emission peak. Importantly, the use of photophysical properties of GQDs could be synergistically implemented with chemotherapeutic treatments, thus increasing the efficacy of the latter. Wang and coworkers used PEG-graphene nanosheets with lateral size comparable to QDs, for multifunctional targeted therapy of malignant glioma [109]. When irradiated by an 808 nm NIR laser (6 W/cm^2^), the solution temperature exceeded 50 °C within 2 min. The photothermal heating exhibited a concentration and laser power intensity-dependent manner. Importantly, they also conjugated a chemotherapeutic agent, doxorubicin, to the nanosheets (Figure 6C). When irradiated, the latter nanocomplex could selectively release the drug, indicating its potential use in a combined therapy.

## 8. GQDs as Drug Delivery Systems

A large amount of research has been done to optimize GQDs’-associated drug delivery systems [110,111,112]. Iannazzo and colleagues designed a new biocompatible and cell traceable drug delivery system, capable of releasing the therapeutic agent doxorubicin to cancer cells in a selective manner [113]. GQDs were covalently linked to biotin, in order to efficiently recognize biotin receptors overexpressed on cancer cells. The chemotherapeutic agent doxorubicin was loaded to the GQD surface, taking advantage of the excellent absorption properties of carbon nanomaterials by $\pi -\pi^{*}$ interaction. Moreover, doxorubicin inherent fluorescence allowed the drug release to be tracked. They measured, by confocal microscopy, an enhanced uptake of the chemotherapeutic agent over time in A549 cells, with a maximum after 6 h; then, it lowered because of increased mortality. Cells treated with doxorubicin-loaded nanoparticles had a delayed mortality with respect to cells treated with free drugs. This could be due to drug detachment from nanoparticles, induced by acid environment of endosomal compartment. In another work, Sui and colleagues used GQDs and cisplatin together to increase the intracellular uptake of the anticancer drug [114]. They experimented on different cell lines and found an increased killing capability for cisplatin coadministered with GQDs when compared to the antitumor alone. The toxicity mechanism of cisplatin is mediated by the cell cycle arrest in G2/M phase. After the treatment with GQDs and the chemotherapeutic, an increase in the population of cells arrested in G2/M was found. Furthermore, another toxicity mechanism of cisplatin at high concentrations involves the formation of apoptotic bodies, significantly increased with GQDs reducing the cisplatin concentration needed. No reduction in the interaction between cisplatin and DNA was measured. Since GQDs enter cells mainly via endocytosis, they could improve cisplatin cellular uptake if transported with GQDs. Indeed, increase of platinum inside cell and nucleus suggested that either GQDs/cisplatin together enter the cells, or GQDs improved the cell permeability at least partially. In our recent work, we tested biocompatible non-toxic GQDs on two neural lineages (U87 human glioblastoma cells and mouse primary cortical neurons) as sensitizing agents for doxorubicin. We used GQDs with three diverse surface chemistries: aminated, carboxylated and non-functionalized. After the treatment with GQDs, we removed the nanoparticles from the medium, and administered doxorubicin, to avoid potentially extracellular interactions between the chemotherapeutic agent and GQDs. Carboxylated and non-functionalized GQDs sensitized U87 cells to doxorubicin, reducing significantly the cell viability. This effect was cell-specific, since it was not visible on neurons, and it was mediated by an increase in membrane permeability of U87 cells due to interactions between GQDs and cell membrane, which was related to the surface net charge of the nanoparticles. The increase in membrane permeability of U87 cells, consequently, increased the uptake of the antitumor drug inside cells with a synergistic mechanism. Wang and colleagues synthesized GQDs conjugated with folic acid to target specifically tumor cells [115]. This nanocomplex was furtherly conjugated with doxorubicin [113]. Release of doxorubicin inside HeLa cells, which overexpress the folic acid receptor was assessed. At first, no release was measured, as indicated by the colocalization of the green channel (GQDs) and the red channel (doxorubicin). Furthermore, to test the specificity of the nanoassembly, they measured cell viability on HeLa cells, A549 and HEK293A. Importantly, the last two cell lines do not express the folic acid receptor. They found a specific reduction in cell viability only for HeLa cells, indicating that GQDs/folic acid conjugated with doxorubicin could release specifically the antitumor drug inside cells overexpressing a folic acid receptor.

**Figure 7:**
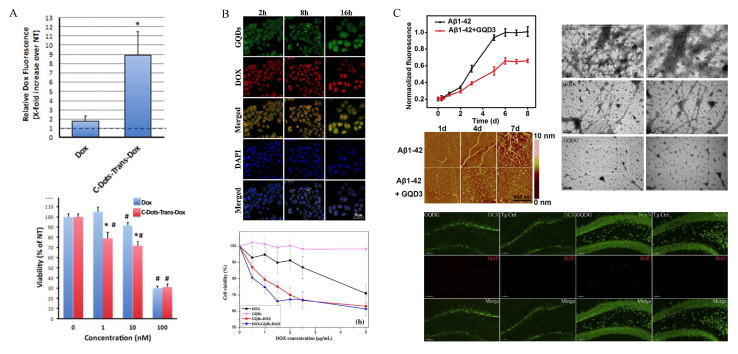
Drug delivery with GQDs. (**A**) Carbon dots functionalized with transferrin and conjugated with doxorubicin. The nanocomplex was administered to SJGBM2 pediatric glioblastoma, resulting in a higher uptake of the chemotherapeutic agent. Adapted with permission from The Royal Society of Chemistry [116]; (**B**) U251 cell treated with GQDs functionalized with a targeting peptide and loaded with doxorubicin. Adapted from [101]. Copyright 2017, permission from Elsevier; (**C**) Neuroprotective activity of GQDs on Alzheimer’s disease, showing a reduction in the fibrillation (top), and the increase in neurogenesis in vivo (bottom, scalebar 60 µm). Reproduced with permission from The Royal Society of Chemistry [117]. Adapted from [118]. Copyright 2016, permission from Elsevier.

Importantly, the ability of GQDs to cross the BBB makes them great candidates for specific drug delivery applications to CNS diseases. Li and Amat covalently attached doxorubicin on a delivery system developed from nontoxic carbon dots and transferrin to transport doxorubicin to pediatric brain tumor cells [116]. The target of transferrin receptors on the BBB enables the transport of chemotherapeutic agents across the BBB and into brain parenchyma. To examine the cellular uptake of the nanocomplex compared to doxorubicin alone, glioma cells were treated with 500 nM conjugate or doxorubicin for 18 h under serum-free conditions. A significant increase in doxorubicin fluorescence was observed by confocal microscopy in the nuclei (Figure 7A). They also tested cell viability on cells treated for 72 h with the nanocomplex compared to cells treated with only doxorubicin. At different concentrations, ranging from 1 to 100 nM, of the nanocomplex, a significant reduction in cell viability was measured.

In another study, Dong and colleagues developed GQDs functionalized with arginine-glycine-aspartic acid to specifically target tumor cells [101]. Doxorubicin was then loaded on the surface of functionalized GQDs and tested on U251 human glioma cells. Cell viability measurements were carried out and, as a result, GQDs conjugated with doxorubicin exhibited a significantly higher lethal effect compared with free doxorubicin (Figure 7B). Most of the released doxorubicin reached nuclei of cells within 8 h of treatment.

Interestingly, GQDs have shown the ability to inhibit the progression of Alzheimer’s disease by reducing the aggregation of β-amyloid (Figure 7C). Liu and colleagues measured a significant reduction in the formation of β-amyloid aggregates caused by the interaction between the negative charges of GQDs and the positive charges of histidine residues on β-amyloid [117]. Subsequently, Xiao and colleagues tested GQDs conjugated with a neuroprotective peptide (glycine-proline-glutamate) [118]. In vitro results showed a significant reduction in the formation of aggregates when β-amyloid were treated with functionalized GQDs (Figure 7C). In vivo studies were then carried out on transgenic mice. To observe β-amyloid associated microglial activation, plaques and activated microglia were labeled on brain slices using immunohistochemistry techniques. A significant reduction in the activation of microglia was pointed out, as well as a reduction in inflammatory factors (such as IL-1 and IL-6) and an increase in the anti-inflammatory factors (IL-4). Another important factor involved in Alzheimer progression is the decrease of dendritic spines, related to the decline of the synaptic plasticity and neuron impairment. Therefore, they measured the density of dendritic spines of neurons in the hippocampus by DiOlistic labeling. Based on the result, they deduced that the synapsis protection effect could come from the reduction of amyloid aggregation. Lastly, they tested neurogenesis by observing an increased number of neural progenitor cells and neurons in mice treated with GQDs, suggesting a possible direct or indirect stimulation of neural proliferation (Figure 7C). Taken together, these studies point out the crucial potential of GQDs as drug delivery systems for CNS pathologies.

## 9. Conclusions

In this review, the current state of the art of GQDs in biomedicine is discussed, with a focus on the neuroscientific field. GQDs are widely expressing their potential in the biomedical world, becoming great candidates for bioimaging and neuroimaging, theranostic and drug delivery applications, especially to the brain, thanks to their ability to cross the BBB. As bioimaging markers, GQDs are currently being employed both in vitro and in vivo, thanks to their great optical properties as well as good biocompatibility. In vitro studies showed that GQDs could easily label different cell lines with high throughput including neurons and neural stem cells. Moreover, some research groups focused on the doping and functionalization of GQDs with different chemical groups. Nitrogen-doped carbon dots were used to label U87 human glioblastoma cells [37], while carboxylated, aminated and DMF-functionalized GQDs labeled C6 glioma cells, resulting only in a low cytotoxicity for DMF-GQDs at relatively high concentrations [38]. Among the in vivo neuroimaging applications described, carbon dots derived from L-aspartic acid and pH-responsive carbon dots were used to selectively label gliomas in tumor-bearing mice [39,96]. GQDs were, and are, also currently being employed for photodynamic and photothermal therapy. U251 human glioma cells were treated with GQDs capable of inducing oxidative stress [102]. As a result, both autophagy and apoptosis pathways were activated, resulting in programmed cell death. Photothermal therapy was applied on nitrogen- and boron-doped GQDs, which were used as in vivo NIR-II imaging probes. Both in vitro and in vivo studies confirmed the photothermal features of doped GQDs, by killing C6 glioma cells and stopping tumor growth [100]. Photothermal therapy was also employed in graphene nanosheets combined with the delivery of doxorubicin [109]. When irradiated, the nanocomplex could selectively release the antitumor drug, indicating its potential in combined therapy. Moreover, as drug delivery systems, some groups used carbon dots conjugated with transferrin and doxorubicin, to efficiently cross the BBB [116].

The capability of interacting with fibrillar proteins of GQDs was exploited to treat Alzheimer’s disease [117,118]. Taken together, these studies unravel the strong potential of GQDs in neuroscience and biomedicine, both in vitro and in vivo, in various techniques involving therapeutic, diagnostic and theranostic applications. To date, GQDs have not undergone clinical trials and clinical translation. Before that, some important challenges must be overcome. First of all, when it comes to administering nanoparticles, spontaneous formation of the biomolecular corona, a non-specific interaction with the proteins in the bloodstream, is inevitable [20]. 

Much effort has been done to control corona formation for graphene-based materials, by surface functionalization with PEG in order to avoid non-specific interactions [27]. A second important challenge relies on the synthesis methods that do not easily allow large scale production of GQD with a reliable and repeatable yield [46]. Biodistribution studies have been performed on in vivo models [67]. However, their limited faithfulness to a human model must be considered before clinical translation. All the effort that has been done on GQDs research has opened to further in vivo applications, in particular for combined therapies involving most of their photophysical features. Even if their properties have been exploited separately, GQDs can serve as optical biomarkers, drug delivery carriers and, along with these features, as photothermal/photodynamic sensors all at once, thus unleashing their full potential, contributing to deepening our knowledge and technology against brain diseases.

## Figures and Tables

**Table 1:** Toxicity of graphene quantum dots (GQDs) and quantum dots (QDs).

Nanoparticles	Functionalization	Cell Line/Animal Model	Exposure Time	Concentration	Tests	Toxicity	Ref.
CdSe QDs	tri-n-octylphosphine oxide capping	Rat hepatocytes	24 h	0.06,0.25,0.1 mg/mL	MTT (viability)	High due to release of Cd atoms	Derfus et al. [19]
CdSe QDs	ZnS capping	Rat hepatocytes	24 h	0.06,0.25,0.1 mg/mL	MTT	Low, no release of Cd (dangerous in vivo)	Derfus et al. [19]
CdSe/ZnS QDs	mercapto-undecanoic acid capping	Vero, h. hepatocytes, HeLa	24 h	From 0.05 to 0.4 mg/mL	CCK-8 (viability)	High at concentrations higher than 0.1 mg/mL	Shiohara et al. [17]
CdSe/CdS QDs	mercaptopropionic acid conjugation	Female Balb/c mice	Up to 15 days	0, 5, 10, 25 mg/kg	NADPH oxidase, LDH, IL-6 (ELISA)	High in spleen, liver, lung and plasma at 25 mg/kg	Haque et al. [60]
GQDs	-	H. leukocytes	36 h	0.2, 0.5 mg/mL	XTT (viability)	Low (10%) at 0.5 mg/mL	Fasbender et al. [61]
GQDs	NH_2_	C. elegans	6 days	From 1 to 100 mg/L	Lethality, movement, neurons assay	Neural damage after chronic exposure	Li et al. [62]
GQDs	NH_2_	Rat alveolar macrophages (NR8383)	24 h	From 0 to 0.5 mg/mL	CCK-8, FITC Annexin V (apoptosis), DNA damage	At high concentrations (0.2 mg/mL)	Xu et al. [63]
GQDs	-, PEG	HeLa, A549	24 h	From 0 to 0.64 mg/mL	WST-1 (viability), ROS, LDH	Low toxicity up to 0.16 mg/mL, no ROS and LDH	Chong et al. [64]
GQDs	NH_2_, COOH, DMF	A549, C6	24 h	From 0 to 0.2 mg/mL	MTT, trypan blue, FITC Annexin V	Low toxicity (viability higher than 80%)	Yuan et al. [38]
GOQDs	FITC	PC12	24 h	From 0 to 0.2 mg/mL	CCK-8, ROS, senescence	No toxicity	Ren et al. [65]
GOQDs	FITC	Zebrafish	24 h	From 0 to 0.2 mg/mL (0.1 for speed and distance)	Mortality, malformation rate, speed	No toxicity	Ren et al. [65]
GQDs	COOH	MDA-MB231	24 h	From 0 to 0.5 mg/mL	MTT, calcein, LDH, FITC Annexin V	Low toxicity (viability around 90%)	Nurunnabi et al. [66]
GQDs	COOH	KB, MDA-MB231, A549, MDCK	24-48 h	From 0 to 0.5 mg/mL	MTT, LDH	Mild toxicity for KB cells in both LDH release and viability (60%)	Nurunnabi et al. [67]
GQDs	COOH	SKH1 female nude mice	22 days	5, 10 mg/kg	Serum biochemistry, histopathological analysis	No histopathological evidences, physiological serum biochemistry	Nurunnabi et al. [67]

**Table 2:** QDs designed to overcome the blood-brain barrier (BBB).

Nanoparticle	Functionalization	BBB Model	Cell Type	Permeability Study	Ref.
Quantum rods	Transferrin conjugation	Transwell	Endothelial cells; astrocytes	Confocal fluorescence imaging	Xu et al. [79]
CdSe/ZnS QDs	MMP-9-siRNA	Single culture	BMVEC	-	Bonoiu et al. [80]
GQDs	-	Transwell	MDCK	Permeability coefficient by fluorescence intensity	Wang et al. [73]
Silica; QDs	NH_2_; COOH; PEG	Transwell	RBT-24H	Permeability coefficient by fluorescence intensity	Hanada et al. [81]
Carbon dots	Nitrogen doping	Transwell	RBMEC; rat astrocytes	Fluorescence intensity of the basolateral medium	Lu et al. [83]
CdSe/ZnS QDs	PEG; NGR peptides	Transwell	BCECs; rat astrocytes; C6	Penetration rate by fluorescence intensity	Huang et al. [84]
CdSe/ZnS QDs	PEG; NGR peptides	Sprague Dawley male rats	-	In vivo imaging; fluorescence intensity of minced tissue	Huang et al. [84]
Carbon Dots	Transferrin conjugation	Zebrafish	-	Confocal fluorescence imaging	Li et al. [78]

**Table 3:** Wavelengths reported in this review for bioimaging, phototherapy and drug delivery on neural cell lineages.

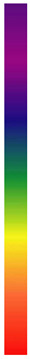	**λ** **(ex, nm)**	**λ** **(em, nm)**	**Nanoparticle**	**Cell Line**	**Ref.**
	360	535	GQDs	hNSCs	Shang et al. [94]
	360	405	N-GQDs	U87	Wang et al. [37]
	360	488	N-GQDs	U87	Wang et al. [37]
	405	540	GQDs	NSCs	Zhang et al. [93]
	405	Blue	Carbon Dots	C6	Zheng et al. [96]
	405	Not available	N-B-GQDs	SF-763	Wang et al. [100]
	405	525	GQDs	U251	Dong et al. [101]
	470	530	GQDs	U251	Markovic et al. [102]
	488	Green	Carbon Dots	C6	Zheng et al. [96]
	488	Not available	N-B-GQDs	SF-763	Wang et al. [100]
	546	Not available	N-B-GQDs	SF-763	Wang et al. [100]
	555	Red	Carbon Dots	C6	Zheng et al. [96]
	Independent	440	GQDs	U87MG	Fan et al. [39]
	Independent	480	GQDs	U87MG	Fan et al. [39]

## Abbreviations

AFM	Atomic Force Microscopy
Asp-CDs	Carbon Dots From L-Aspartic Acid
ATP	Adenosine 5′-triphosphate Disodium Salt
BBB	Blood-Brain Barrier
C_60_	Fullerene
CA	Citric Acid
CCK-8	Cell Counting Kit-8
CNS	Central Nervous System
DAPI	4′,6-Diamidino-2-Phenylindole
DMF	Dimethylformamide
EDTA	Ethylene Diamine Tetraacetic Acid
ELISA	Enzyme-Linked Immunosorbent Assay
FITC	Fluorescein Isthiocyanate
GO	Graphene Oxide
GQDs	Graphene Quantum Dots
HOMO	Highest Occupied Molecular Orbital
IL	Interleukin
LDH	Lactate Dehydrogenase
LUMO	Lowest Unoccupied Molecular Orbital
MPP+	1-Methyl-4-phenyl-pyridinium ion
MTT	3-(4,5-Dimethylthiazol-2-yl)-2,5-diphenyltetrazolium bromide
NADPH	Nicotinamide adenine dinucleotide phosphate
NIR	Near-Infrared Femtosecond Laser Excitation
NIR-II	Second Near-Infrared Window Laser Excitation
P_app_	Apparent Permeability Coefficient
PDT	Photodynamic Therapy
PEG	Polyethylene Glycol
PL	Photoluminescence
PTT	Photothermal Therapy
QDs	Quantum Dots
ROS	Reactive Oxygen Species
SiRNA	Short-interfering RNA
TABT	1,3,5-Tri-amino-2,4,6-trinitrobenzene
TEER	Trans-Endothelial Electrical Resistance
WST-1	Water Soluble Tetrazolium Salt-1
XTT	2,3-Bis-(2-methoxy-4-nitro-5-sulfophenyl)-2H-tetrazolium-5-carboxanilide
